# 2820. Clinical Outcomes in Patients Who Receive a One-Time Aminoglycoside Dose for ESBL Enterobacterales or *Pseudomonas aeruginosa* cystitis

**DOI:** 10.1093/ofid/ofad500.2431

**Published:** 2023-11-27

**Authors:** Kelsey Bouwman, Melissa George

**Affiliations:** ECU Health Medical Center, Greenville, North Carolina; ECU Health Medical Center, Greenville, North Carolina

## Abstract

**Background:**

Extended spectrum beta lactamase (ESBL) Enterobacterales and *Pseudomonas aeruginosa* can be highly resistant organisms resulting in prolonged hospitalizations or the requirement of outpatient parenteral antibiotics. The Infectious Disease Society of America recommends a single dose of an aminoglycoside for uncomplicated cystitis caused by ESBL Enterobacterales and difficult to treat *Pseudomonas aeruginosa*. However, there is very little recent clinical evidence to support this recommendation. At our institution, we extrapolate this data to complicated cystitis and recommend a one-time aminoglycoside dose following about 3 days of effective therapy to help facilitate completion of antibiotic therapy and/or discharge. The objective of this study was to evaluate the safety and efficacy of a single-dose aminoglycoside for cystitis caused by ESBL Enterobacterales or *Pseudomonas aeruginosa*.

**Methods:**

This was a multicenter, retrospective, cohort study. Adult patients treated for cystitis with ESBL Enterobacterales or *Pseudomonas aeruginosa* from January 2020 to December 2022 were identified for analysis, and data was obtained from electronic health records. Patients who received ≥ 3 days of standard of care were compared to patients who received a one-time dose of an aminoglycoside with or without a short course of effective therapy before. The primary outcome of this study was rate of relapse, and secondary outcomes included readmission rates, length of stay, and safety.

**Results:**

A total of 66 patients were included in the study, with 33 patients in each study arm. The study populations were comparable except there were more males and complicated cystitis patients in the standard of care group. There was no difference found in the rate of relapse. The length of stay was significantly different between the two groups (4.5 ± 4.4 days in the aminoglycoside group vs 14.1 ± 10.1 days in the standard of care group, p < 0.0001). No difference was found between the groups for readmission or safety.

**Conclusion:**

A one-time dose of an aminoglycoside did not increase risk of relapse and was associated with shorter length of stay when used to treat cystitis caused by ESBL Enterobacterales or *Pseudomonas aeruginosa*.

**Disclosures:**

**All Authors**: No reported disclosures

